# Left atrial appendage occlusion: are we ready for prime time?

**DOI:** 10.1093/europace/euae187

**Published:** 2024-07-31

**Authors:** Jacopo Marazzato, Luigi Di Biase

**Affiliations:** Montefiore Medical Center, Albert Einstein College of Medicine, 111 East 210th Street, Bronx, NY 10467, USA; Electrophysiology and Cardiac Pacing Unit, Humanitas Mater Domini, Castellanza, Italy; Montefiore Medical Center, Albert Einstein College of Medicine, 111 East 210th Street, Bronx, NY 10467, USA

**This editorial refers to ‘Incidence and predictors of 2-year mortality following percutaneous left atrial appendage occlusion in the EWOLUTION trial’ by E.W. Aarnink *et al*., https://doi.org/10.1093/europace/euae188.**

Since atrial fibrillation (AF) is associated with a five-fold increased risk of ischaemic stroke, oral anticoagulation therapy (OAT) is mandatory in AF patients with a high thromboembolic profile.^1,2^ However, the increasing age and burden of comorbidities may preclude these patients from assuming OAT for an excessive haemorrhagic hazard. Given that most of AF-related thrombi stem from the left atrial appendage (LAA),^3^ a variety of surgical, percutaneous (Amplatzer^™^ Amulet^™^ and WATCHMAN^™^), and hybrid (LARIAT^™^) techniques for LAA occlusion (LAAO) have been developed over the years^3–5^ to protect this frail population against the detrimental effects of stroke and systemic embolism.

The WATCHMAN system is the most investigated percutaneous LAAO (pLAAO) device with Food and Drug Administration approval.^3^ Randomized controlled trials (RCTs)^6–8^ showed that WATCHMAN was non-inferior to vitamin K antagonists^6,7^ and direct oral anticoagulants (DOACs)^8^ for stroke prevention and was superior for the reduction of the haemorrhagic hazard even at a long-term follow-up.^9^ Hence, European and American guidelines^1,2,4^ now recommend pLAAO in non-valvular AF patients with OAT contraindication (Class IIa^2^ and IIb,^1^ level of evidence B) or in those deemed at high bleeding risk (Class IIb, level of evidence B^2^).

Although the strength of these recommendations is weakened by the exclusion of AF patients with OAT contraindication from the WATCHMAN trials, non-randomized studies helped us understand the feasibility of WATCHMAN in these cases.^10–12^

The Evaluating Real-Life Clinical Outcomes in Atrial Fibrillation Patients Receiving the WATCHMAN Left Atrial Appendage Closure Technology (EWOLUTION) study is the largest prospective, real-world registry to date in this setting. The trial was conducted on 1020 non-valvular AF patients undergoing WATCHMAN implantation for stroke prevention and with OAT contraindication. Compared with a historic patient cohort off OAT and with a similar risk profile, WATCHMAN was associated with a significant 2-year risk reduction of the ischaemic and haemorrhagic hazard.^12^ However, due the advanced age and the burden of comorbidities of this population, the reported 1- and 2-year all-cause mortality was remarkably high, 9.8% and 16.4%, respectively.^11,12^

Therefore, the pre-procedural identification of clinical variables that may potentially influence patient’s survival post-pLAAO is paramount for ensuring the feasibility of this strategy for stroke prevention in this high-risk population.

In this issue of the journal, Aarnink *et al*.^13^ identified the predictors of 2-year mortality in non-valvular AF patients enrolled in the EWOLUTION registry. In a multivariate analysis, age and cardiovascular and non-cardiovascular comorbidities not only proved independent predictors of all-cause mortality after pLAAO with WATCHMAN, but their combination in a risk score also showed a different mortality hazard ranging from 5.6–5.7 to 46.1% when multiple risk factors were considered. In addition, death mechanism was clarified since most of the deceased patients died of non-cardiovascular causes out of which major haemorrhagic events represented 20% of cases.

Although this study is affected by several limitations—lack of randomization and of standardized definitions of the evaluated risk factors, the unclear contraindications for OAT, and no validation of the proposed risk score—it should be nonetheless regarded as the first evidence providing guidance in the identification of which AF patients with OAT contraindication might benefit from pLAAO for stroke prevention.

However, other not appraised peri- and post-procedural factors might have had an impact on the 2-year all-cause mortality rate observed by Aarnink *et al*.^13^

First, the significant peri-procedural complication hazard—3.3%^11^—might partly explain the high mortality risk recorded in this frail population. In fact, in the EWOLUTION registry, patients were enrolled during the first days of WATCHMAN when it was regarded as a last resort of non-valvular AF patients with OAT contraindication. However, the rate of peri-procedural complications post-WATCHMAN implantation has progressively decreased over the years, from the 8.7% in the ASAP study^10^ to the 3.2% of the PROTECT AF,^6^ the 2.2% of the PREVAIL,^7^ and, finally, as low as the 0.5% of the PINNACLE FLEX trial,^14^ thereby suggesting a better prognosis in patients with new generation devices.^14^

Second, current antithrombotic therapies post-WATCHMAN implantation are based on different empirical schemes tested in RCTs evaluating patients without OAT contraindication.^4^ Therefore, a wide range of non-standardized antithrombotic regimens might have influenced the dismal prognosis observed in the EWOLUTION study^12^ with antiplatelet regimens carrying a worse prognosis when compared with OAT. Likewise, recent evidence proved that long-term half-dose DOACs led to significantly lower embolic and haemorrhagic risks after successful pLAAO with WATCHMAN, thereby suggesting the limited and potentially hazardous role of antiplatelet therapy over OAT in this setting.^4^

Finally, a Swedish cost-effectiveness analysis of these patients showed that pLAAO not only improved the quality of life of these high-risk cases when compared with no pharmacological treatment but, from a public sector perspective, also reduced the long-term costs for home care, thanks to the substantial abatement of the stroke clinical sequelae.^15^

Therefore, are we ready for prime time for pLAAO in all AF patients? First, in high-risk cases, patient selection is mandatory to increase the cost-effectiveness and to reduce the mortality risk related to the procedure. In this regard, the ASAP-TOO (assessment of the WATCHMAN device in patients unsuitable for oral anticoagulation) trial (NCT02928497) will also help us choose the best antithrombotic strategy post-WATCHMAN implantation in these cases. Second, to a further extent, the CATALYST (Amplatzer Amulet LAAO vs. DOAC) trial (NCT04226547), the CHAMPION-AF (WATCHMAN FLX vs. DOAC for embolic protection management of patients with non-valvular AF) trial (NCT04394546), and the OPTION (comparison of anticoagulation with LAA closure after AF ablation) trial (NCT03795298) will help clarify whether pLAAO might represent an actual alternative to OAT in all subsets of patients with AF.

For the time being, pLAAO should be regarded as the only OAT alternative that can prevent the troublesome consequences of stroke in high-risk patients with OAT contraindication.
